# Postscript

**Published:** 1895-08

**Authors:** 


### POSTSCRIPT.

Sometime ago I had an opportunity to verify the action of *Cundurango* in ulceration of the lip. A boy of about eighteen came to the office with an irregular, rather deep ulcer on the upper lip, right side, and without other symptoms upon which to base a prescription. One dose of Cundurango$^{10m}$ (F.), entirely cured the ulcer without other help. It is folly to make local applications or to use the knife in such cases. The remedy is ever efficient, and sometimes the location forms the keynote for a condition of this kind, though it is not wise to rely on it when we can find symptoms upon which to base a prescription.

The peculiar symptom, "Sensation of something flapping back and forth in the urethra, with itching, soreness, and cutting after urination, and swelling of urethra" (HOMŒOPATHIC PHYSICIAN, Vol. XIV, page 399), led me in a very complicated case to the remedy. It was such a case that I would not have been able to find the remedy without some trouble, but with the aid of this symptom I was led direct to *Argentum-nitricum.* The remedy in the CM potency, the single dose, acted promptly and satisfactorily.

Such peculiar symptoms are valuable as indices to the materia medica and often save very much time and labor. It is needless to say to the readers of *The Organon* that no prescription should be based on a single indication when it is possible to get more, and that no prescription should be made without first verifying the choice of the remedy by the materia medica. It is very seldom I give a remedy without first looking into the pathogenesis of the drug. The patients like to see their doctor careful. Once on the second prescription I gave Sac-lac. without opening a book, as the case was doing so well, but the lady came back in a couple of days, saying she was fearful I had been in too big a hurry and had not done my best, "*as I had not looked into the books.*" One must *look* as wise in giving Sac-lac. as in giving a true remedy.

